# Evaluation of an Objective Measurement Tool for Stress Level Reduction by Individually Chosen Music During Colonoscopy—Results From the Study “ColoRelaxTone”

**DOI:** 10.3389/fmed.2020.00525

**Published:** 2020-09-15

**Authors:** Steffen Walter, Sascha Gruss, Jana Neidlinger, Isabelle Stross, Alexander Hann, Martin Wagner, Thomas Seufferlein, Benjamin Walter

**Affiliations:** ^1^Sektion Medizinische Psychologie, Klinik für Psychosomatische Medizin und Psychotherapie, Universitätsklinik Ulm, Ulm, Germany; ^2^Klinik für Innere Medizin I, Universitätsklinik Ulm, Ulm, Germany; ^3^Medizinische Klinik und Polyklinik II, Universitätsklinik Würzburg, Würzburg, Germany

**Keywords:** colonoscopy, anxiety, stress level, music, relaxation

## Abstract

**Background and Aims:** Colonoscopy as standard procedure in endoscopy is often perceived as uncomfortable for patients. Patient's anxiety is therefore a significant issue, which often lead to avoidance of participation of relevant examinations as CRC-screening. Non-pharmacological anxiety management interventions such as music might contribute to relaxation in the phase prior and during endoscopy. Although music's anxiolytic effects have been reported previously, no objective measurement of stress level reduction has been reported yet. Focus of this study was to evaluate the objective measurement of the state of relaxation in patients undergoing colonoscopy.

**Methods:** Prospective study (*n* = 196) performed at one endoscopic high-volume center. Standard colonoscopy was performed in control group. Interventional group received additionally self-chosen music over earphones. Facial Electromyography (fEMG) activity was obtained. Clinician Satisfaction with Sedation Instrument (CSSI) and Patients Satisfaction with Sedation Instrument (PSSI) was answered by colonoscopists and patients, respectively. Overall satisfaction with music accompanied colonoscopy was obtained if applicable.

**Results:** Mean difference measured by fEMG via musculus zygomaticus major indicated a significantly lower stress level in the music group [7.700(±5.560) μV vs. 4.820(±3.330) μV; *p* = 0.001]. Clinician satisfaction was significantly higher with patients listening to music [82.69(±15.04) vs. 87.3(±15.02) pts.; *p* = 0.001]. Patient's satisfaction was higher but did not differ significantly.

**Conclusions:** We conclude that self-chosen music contributes objectively to a reduced stress level for patients and therefore subjectively perceived satisfaction for endoscopists. Therefore, music should be considered as a non-pharmacological treatment method of distress reduction especially in the beginning of endoscopic procedures.

## Introduction

Colonoscopy is a most common procedure and gold standard used in the diagnosis and treatment of diseases in the lower gastrointestinal tract (1). However, endoscopy is perceived as stressful for many patients due to anxiety, discomfort, pain or previous unpleasant experience. Patient's anxiety regarding colonoscopy may result from strangeness of the hospital environment, a lack of knowledge regarding the endoscopic procedure, the uncertainty of outcome, fear of pain and worry about the recovery process (2–4). High levels of anxiety and pain may result in a more difficult procedure, increased need for sedatives, and therefore increased likelihood of medication related complications, low patient satisfaction and risk for incomplete examination (5). A variety of interventions have been attempted to reduce the level of stress prior and during endoscopy. Pharmacological approaches by sedatives and analgesic drugs are often offered, although their use is associated with increased risks and costs, as mentioned above. Non-pharmacological attempts such as supplemental reading, images of nature or other scenes were previously evaluated (6). As an inexpensive, safe, and effective non-pharmacological anxiolytic, music has historically been used as a nursing intervention to relieve illness and suffering, taking away patients' attention from the negative experience and replacing it with encouraging thoughts. Thus, it is reasonable to offer this non-invasive modality to patients during colonoscopy. The use of music is already recommended by national, European and international guidelines but with up to now poor evidence (5, 7, 8). Previous studies have examined the impact of music during various endoscopic procedures, but the results remain inconclusive (9–16). A major limitation is the lack of objective measurement of patient satisfaction after propofol sedation due to the known euphoric effect of the agent. Vital signs such as heart rate, respiratory rate, blood pressure and oxygen saturation are available but results may be jeopardized by co-medication like anti-hypertensives (14). Secondly, the choice of music is limited by the music offerings available in the endoscopy unit. Individual and specific musical taste should also be taken into account. So, music which is perceived as relaxing by the patient might not trigger this effect for the endoscopy staff and vice versa. Therefore, it should be considered to play music chosen by the patient himself over headphones. The aim of this prospective study was to evaluate the impact of patient selected music on stress reaction to colonoscopy and the feasibility of using easy implementable objective physiologic stress measurement tools.

## Methods

A prospective study was conducted at an endoscopic unit at the university hospital of Ulm, Germany between November 2019 and February 2020. Individuals (≥18 yrs.) with a scheduled out- or inpatient colonoscopy were enrolled. In case of impaired mental status, severely compromised medical status; deafness, American Society of Anaesthesiologists (ASA) risk class III and higher; pregnancy and declined participation, individuals were excluded from the trial. Written informed consent was obtained from all patients.

Complete datasets from 196 patients were available. Ninety eight patients were examined who did not listen to music (sample 1) during treatment and 98 patients who listened to music (sample 2). The time difference from the previous sample 1 (last patient: 01.07.19, NCT:03860779, “Biopotentials for Clinician Satisfaction with Sedation in Colonoscopy”) vs. sample 2 (first patient: 12.11.19, NCT:04258800) was about 18 weeks. Based on the prior preliminary results on this topic we calculated the power of an low effect size of 0.25.

### Colonoscopy

All patients received standard bowel preparation by split-dose PEG-based preparation regime. Low fiber-diet was recommended for 3 days before colonoscopy. The colonoscopies were performed according to national endoscopy guidelines (8). Single propofol boluses were performed in order to reach the target sedation level. Insertion of colonoscopy was started simultaneously with the first administration of propofol. All colonoscopies were performed by experienced endoscopists (experience with at least >1,000 colonoscopies). Overall, 7 endoscopists participated in the study who were not aware of the study goal! Standard monitoring (blood pressure, heart rate, oxygen saturation) for endoscopic procedures was carried out in accordance with endoscopy guidelines.

### Assessments

The demographic data, including age, gender, and body mass index (BMI) were collected. Further variables known to influence the anxiety and pain level as history of prior colonoscopy, history of abdominopelvic surgery and comorbidities were assessed upon study allocation. Patients in the control group received standard care. Patients in the interventions group received standard care and were additionally asked to choose their favorite music on a music streaming platform (Spotify, Spotify Technology S.A., Sweden) provided by the study nurse. Patients were equipped with adjustable special on-earphones for sleeping, directly upon arrival in the examination room. They consisted of high-quality audio speakers nestled inside a disinfectable, wearable headband to avoid any discomfort when lying in left lateral position for endoscopy (Sleep Headphones Bluetooth Sport Headband, Lavince, China). Music was transmitted wirelessly. Volume was controlled by the patients. Immediately after the colonoscopy the music was stopped and the earphones were removed by the study nurse.

### Questionnaires

All examiners were asked to fill out the Clinician Satisfaction with Sedation Instrument (CSSI), directly after the examination (17). Additionally, patients filled out the Patients Satisfaction with Sedation Instrument (PSSI) before discharge in the outpatient setting and on the ward in case of an inpatient treatment. Satisfaction with listening to music during colonoscopy was obtained by an 8-item satisfaction questionnaire if applicable.

### Measured Physiological Parameters

The following biophysiological signals of the autonomic nervous system (B-ANS) were recorded at the rate of 2,048 Hz, utilizing a Nexus-amplifier and the corresponding BioTrace-Software (www.mindmedia.com).

**Figure 1:**
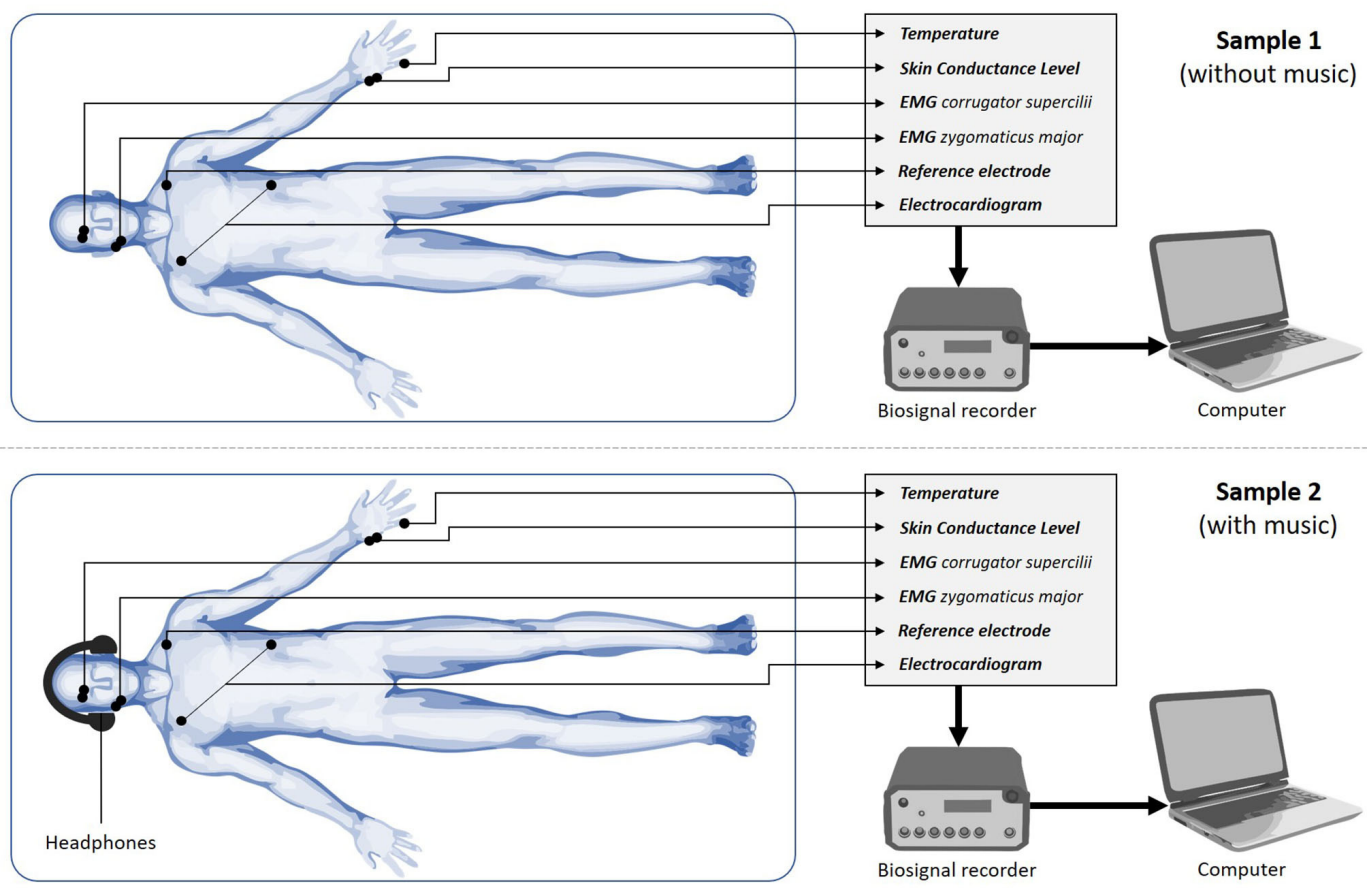
Study setup.

Bipolar pairs of Ag/AgCl electrodes were utilized for measuring Facial Electromyography (fEMG) activity (18–22). The electrodes were placed over the right *corrugator supercilii* and right *zygomaticus major* muscles (Figure 1).

Three single Ag/AgCl electrodes were utilized to measure the average cardiac action potential by electrocardiogram (ECG) on the skin. One electrode was placed on the chest, ~6 cm below the right collarbone. The second electrode was placed on the left lower rib cage. The third electrode served as reference and was attached to the right-side waist next to the pelvic bone. It also served as reference for the fEMG. Two electrodes were placed on the edge of the left hand to measure electrodermal activity via skin conductance level (SCL). A temperature (TMP) sensor was attached to the tip of the left little finger with a medical tape.

### Biosignal Processing and Feature Extraction

To speed-up processing, all recorded biosignals were downsampled to 512 Hz. Afterwards all signals were individually processed: fEMG: A band-pass filter (Butterworth design, 20–250 Hz) was applied to filter the raw fEMG data. Next, a Hilbert-transformation was performed and absolute values of the resulted signal were obtained. Finally, the data were low-pass filtered with a cut-off frequency of 4 Hz. SCL: The SCL signals were low-pass filtered with a cut-off frequency of 8 Hz. ECG and TMP: A moving average windows (for ECG: *n* = 67; for TMP: *n* = 513) was used to smoothen the data. Additionally, all ECG signals were detrended. For statistical calculations, 1 min windows were cut out of the signals at the following time points: “*Examination start*” (60 s right after the beginning of the examination), “*Coecum*” (30 s before plus 30 s after reaching the cecum) and “*Examination end*” (60 s before the examination ended). A visual inspection was carried out to identify corrupted segments. Artifacts and outliers were corrected where possible, otherwise the whole window was discarded. In a final step, relevant features for all signals from each 1 min window were derived: For ECG: R-peaks were detected with the QRS-detection algorithm by Hamilton and Tompkins and used to calculate heart beats per minute. For fEMG, SCL, and TMP: The mean value for each window was determined (for fEMG in μ*V*, for SCL in μ*S* and for TMP in °*C*).

### Statistical Analysis

Statistical tests were performed using SPSS Statistics 25 (IBM, USA). A non-parametric comparison between the groups “without music” vs. “with music” was calculated (U-test) via “*Examination start*,” “*Coecum*,” and “*Examination end*.” The chi-square test, Fisher's exact test, student's *t*-test, and correlation analysis were used wherever applicable. A *p* < 0.05 indicated statistical significance.

## Results

Patient characteristics can be seen in Table 1. No significant differences were found between the two groups.

**Table 1:** Demographic and clinical characteristics of the participants; SD, Standard deviation; ASA, American Society of Anaesthesiologists; BMI, Body mass index; BBPS, Boston bowel preparation scale; CRC, Colorectal Cancer; IBD, inflammatory bowel disease.

**Characteristic**	**Control (*n* = 98)**	**Music (*n* = 98)**	***p*-value**
Years, mean (±SD)	53.62 ± 17.29	55.16 ± 15.02	0.627^b^
Sex, *n* (%) Male Female	51 (52) 47 (48)	50 (51) 48 (49)	0.886^a^
BMI, mean (±SD), kg/m^2^	25.68 ± 4.70	25.78 ± 4.93	0.719^b^
Colonoscopy, *n* (%) Outpatient Inpatient	65 (66) 33 (34)	68 (69) 30 (31)	0.646^a^
Propofol, mg (±SD)	208.00 ± 97.32	224.29 ± 108.39	0.288^b^
BBPS, pts (±SD)	6.36 ± 1.80	6.63 ±1.73	0.247^b^
Cecal intubation rate, *n* (%) Yes (coecum reached) No (coecum not reached) Not specified	92 5 1	92 4 2	0.745^a^
Withdrawal, min (±SD)	8.93 ± 4.54	9.74 ± 4.08	0.088^b^
Abdominal surgery, *n* (%) No Yes No information	88 (90) 10 (10)	84 (86) 13 (13) 1 (1)	0.489^a^
Indication, *n* (%) CRC screening/surveillance IBD Abdominal complaints Anemia Others No information	24 (25) 30 (31) 23 (24) 11 (11) 8 (8) 2 (2)	28 (29) 24 (25) 23 (24) 11 (11) 8 (8) 4 (4)	0.896^a^
Co-morbidities, *n* (%) IBD Diabetes mellitus Hypertension Thyroid disease Chronic kidney disease	28 (29) 7 (7) 27 (29) 19 (20) 7 (8)	24 (25) 13 (14) 33 (35) 18 (19) 7 (7)	0.490^a^ 0.148^a^ 0.348^a^ 0.883^a^ 0.983^a^
Prior Colonsocopies, *n* (%) Yes No No information	64 15 19	75 22 1	0.550^a^

a *Chi Quadrat Pearson*;

b *Mann Whitney-U*.

**Figure 2:**
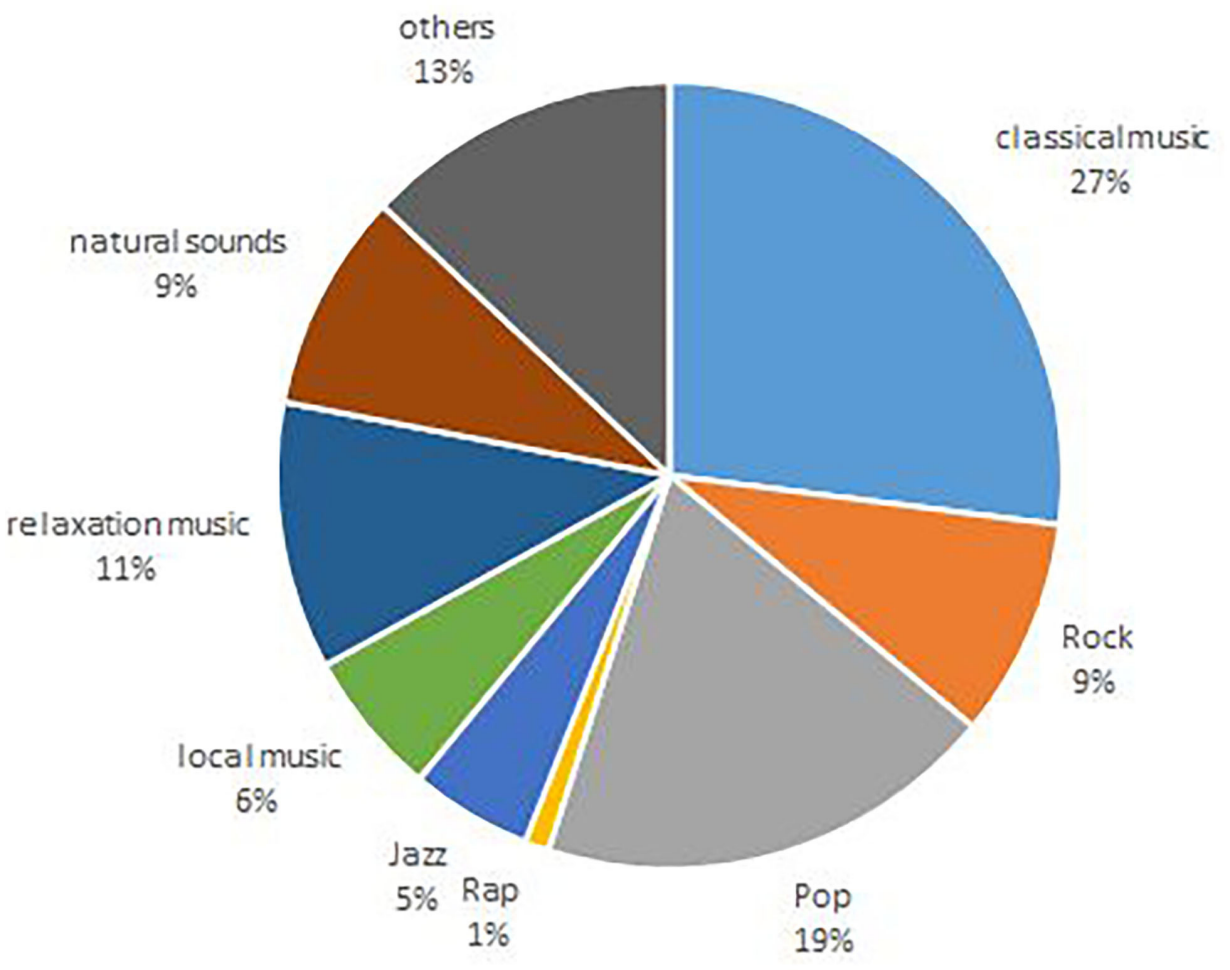
Music styles chosen by participants.

Figure 2 shows the heterogeneous music styles chosen by the participants. Table 2 shows results of the comparisons between CSSI and PSSI. The one-sided comparison of CSSI and PSSI, is significant (*p* = 0.004) only for the CSSI. This means that the difference of the observers' satisfaction score has increased from 82.69 to 87.30 points in the examinations with music. The effect is in the expected direction for both sexes, but when viewed separately, only female show a significant difference between sample 1 vs. sample 2 (*p* = 0.002). The fact that there is no significant difference between the mean value comparisons of the PSSI might be probably due to a ceiling effect, since in the sample without music, the PSSI is clearly >90%.

**Table 2:** Mean value difference for sample “without music” (sample 1, *n* = 98) vs. sample “with music” (sample 2, *n* = 98) via CSSI and PSSI (*significant one side *p* ≤ 0.05).

**CSSI**					**PSSI**				
**Number of patients**	***N***	**Mean**	**SD**	***p*-level (one-side)**	**Number of patients**	***N***	**Mean**	**SD**	***p*-level (one-side)**
Sample 1	98	82.69	15.04	0.004*	Sample 1	78	93.38	11.44	0.477
Sample 2	94	87.30	15.02		Sample 2	96	92.67	9.85	
**Female**					**Female**				
Sample 1	47	79.79	15.12	0.002*	Sample 1	36	91.09	14.71	0.895
Sample 2	47	87.09	16.41		Sample 2	47	93.03	9.22	
**Male**					**Male**				
Sample 1	51	85.36	14.61	0.441	Sample 1	42	95.34	7.26	0.270
Sample 2	47	87.50	13.67		Sample 2	49	92.32	10.50	

**Figure 3:**
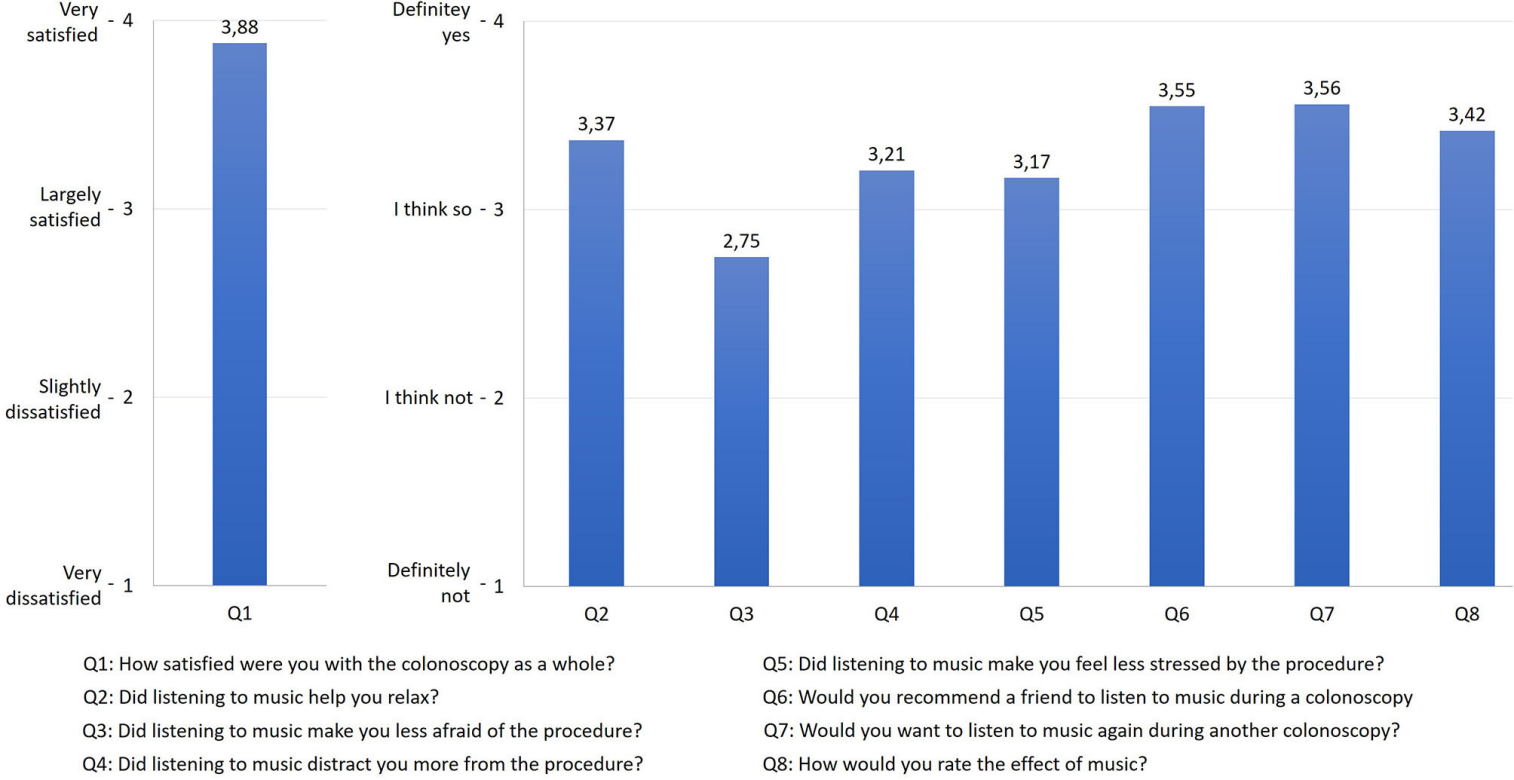
Subjective evaluation in terms of the subjective perception of musical taste.

Figure 3 shows an evaluation in terms of the subjective perception of music during colonoscopy. Descriptively, it can be concluded that patients find listening to music pleasant and relaxing. The impact of fear reduction was less compared to the other parameters. Listening to music would potentially be further recommended. It is essential that patients who already had a colonoscopy found the procedure more tolerable with music.

Tables 3, 4 present the results of the comparison “without” vs. “with music” via fEMG activity.

**Table 3:** Mean value difference for sample “without music” (sample 1, *n* = 98) vs. sample “with music” (sample 2, *n* = 98) via facial electromyography [fEMG] of *corrugator supercilii* in μV (*significant one side *p* ≤ 0.05).

		**Examination start**			**Coecum**			**Examination end**		
**Number of patients**	***N***	**Mean**	**SD**	***p*-level (one-side)**	**Mean**	**SD**	***p*-level (one-side)**	**Mean**	**SD**	***p*-level (one-side)**
Sample 1	64	8.15	7.72	0.017^*^	6.86	5.46	0.729	7.23	5.87	0.463
Sample 2	82	7.36	7.13		8.06	8.06		7.24	6.40	
**Female**										
Sample 1	30	8.12	7.57	0.287	8.59	7.10	0.971	8.15	6.41	0.707
Sample 2	39	7.68	7.08		9.80	9.32		7.68	6.28	
**Male**										
Sample 1	34	8.17	7.96	0.029^*^	5.33	2.72	0.511	6.42	5.31	0.454
Sample 2	43	7.08	7.24		6.49	6.42		6.84	6.56	

**Table 4:** Mean value difference for sample “without music” (sample 1, *n* = 98) vs. sample “with music” (sample 2, *n* = 98) via facial electromyography [fEMG] of *zygomaticus major* in μV (*significant one side *p* ≤ 0.05).

		**Examination start**			**Coecum**			**Examination end**		
**Number of patients**	***N***	**Mean**	**SD**	***p*-level (one-side)**	**Mean**	**SD**	***p*-level (one-side)**	**Mean**	**SD**	***p*-level (one-side)**
Sample 1	65	7.70	5.56	0.001^*^	4.82	3.42	0.001^*^	5.17	3.8	0.470
Sample 2	84	4.82	3.33		3.70	3.27		4.30	3.03	
**Female**										
Sample 1	31	5.85	2.43	0.027^*^	4.28	1.85	0.096	4.85	2.82	0.397
Sample 2	41	4.90	2.92		4.36	4.42		4.58	3.29	
**Male**										
Sample 1	34	9.39	6.97	0.001^*^	5.32	4.37	0.001^*^	5.46	4.52	0.026^*^
Sample 2	43	4.75	3.71		3.06	1.28		4.02	2.76	

**Figure 4:**
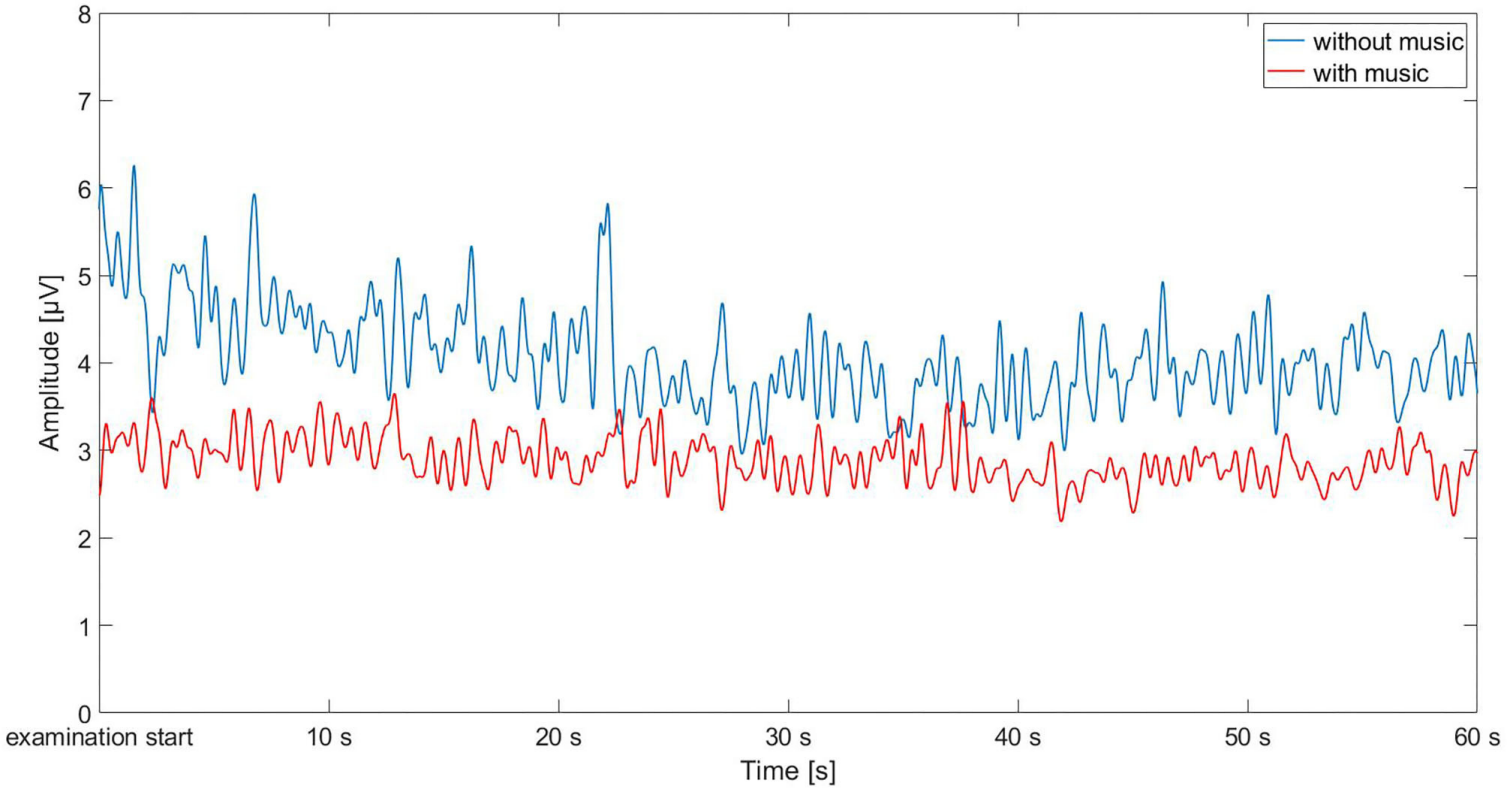
Examination start via facial electromyography [fEMG] of *zygomaticus major* in μV, exemplary for a patient with music and a patient without music.

In particular, the fEMGs of the *corrugator supercilii* and *zygomaticus major* muscles at the beginning of the examination demonstrate decreased fEMG stress level (regardless of sex) (Figure 4). This is in the line with former results via stress level of affective computing and automated pain recognition and regarding biofeedback (18, 21).

However, the effect of the corrugator regarding women is not significant.

The results of the SCL, TMP, and ECG show no evidence of reduced sympathicus activity during listening to music.

## Discussion

Distress and pain are common issues in endoscopy. Especially colonoscopy is subjectively associated with emotional distress and physical discomfort. Non-pharmacological approaches are mandatory to reduce distress and pain in patients to avoid increased need for sedatives. In this field, music has the capacity of adjusting emotional stats and promotes mind-body interactions widely accepted for promoting relaxation in health care. In addition, there are many old and new findings that distraction reduces the distress or pain during medical treatments (10, 23). In our study, patients who listened to music reported a high level of satisfaction with the endoscopy. These results are consistent with data obtained in previous studies (16). Moreover, most of the participants indicated that they would want to have music available again during a future colonoscopy.

It might be interesting pointing out that self-selected music of the patient has a positive influence on the overall evaluation of the treatment for both clinicians and patients.

Objective measurement of stress level of the patients is mandatory as deductive methods are most likely to be biased. Especially heart rate is affected by multiple factors which might jeopardize the impact of stress modifying non-pharmacological agents. Numerous findings have shown that measuring electromyography in the face, is a highly sensitive measure of distress activity and pain (18, 20, 24, 25). The reduction of the fEMG activity is most evident at the beginning of the treatment. This is might be a hint as the patient still has conscious or limited conscious perception in this phase and in this respect the music has a stress-reducing effect. This is in line with the patients' subjective reflection, as they can only evaluate subjectively what they have consciously perceived.

Regardless of focusing the elicitation of music and distress response under the context of a colonoscopy, the fEMG could be an easy implementable tool for monitoring during colonoscopy.

The fact that there are no effects in both SCL and ECG could be due to the context that this is highly individual-specific and that sample 1 and 2 were independent of each other. The results of the TMP cannot be clearly interpreted.

Negative feelings not only cause negative physiological manifestations, but can also increase the complications of medication used for sedation. Contrary to previous data music revealed a significant impact on only one physiological parameter (fEMG) in our study. This may be due to the individual responding to music. The effect might be enhanced by the participants' musical preferences.

We acknowledge several limitations in this study. Firstly, due to its monocentric design general transferability might be impaired. Future studies should demonstrate the impact of self-chosen music on objective measurement of distress and pain levels in patients undergoing colonoscopy. Secondly, due to the limited number of patients the impact of the music genre chosen by individuals remains unclear.

Further studies should examine timing of music start, role of ambient music and elaborate a general recommendation for music with relaxing content for both patients and endoscopy staff.

## Conclusion

In summary, these findings demonstrate that self-chosen music provides an objectively measurable benefit for patients and clinicians in the setting of colonoscopy. The use of music can be recommended as an adjuvant to enhance patient's experience and support a higher participation in e.g., CRC screening.

## Data Availability Statement

We are willing to share the data set in cooperation with other working groups. Requests to access the datasets should be directed to Steffen Walter, steffen.walter@uni-ulm.de.

## Ethics Statement

The study was approved by the Ethics Commission in Ulm. The patients/participants provided their written informed consent to participate in this study.

## Author Contributions

SW: literature search, data analysis, and writing. SG: study design, data analysis, and writing. JN and IS: data collection. MW and TS: critical revision. BW: literature search, study design, interpretation, and writing. AH: critical revision and data collection. All authors contributed to the article and approved the submitted version.

## Conflict of Interest

The authors declare that the research was conducted in the absence of any commercial or financial relationships that could be construed as a potential conflict of interest.

## Abbreviations

B-ANS: Biophysiological signals of the autonomic nervous system
BBPS: Boston bowel preparation scale
BMI: Body Mass Index
CRC: Colorectal cancer
CSSI: Clinician Satisfaction with Sedation Instrument
ECG: Electrocardiogram
fEMG: Facial electromyography
IBD: Inflammatory bowel disease
PSSI: Patient Satisfaction with Sedation Instrument
SCL: Skin conductance level
TMP: Temperature.
